# Mechanical Comparison of a Novel Hybrid and Commercial Dorsal Double Plating for Distal Radius Fracture: In Vitro Fatigue Four-Point Bending and Biomechanical Testing

**DOI:** 10.3390/ma14206189

**Published:** 2021-10-18

**Authors:** Hsuan-Chih Liu, Yu-Hui Zeng, Chun-Li Lin

**Affiliations:** 1 Division of Orthopedics, Chi Mei Hospital, Liouying, Tainan City 710, Taiwan; mvphoton@gmail.com; 2 Department of Biomedical Engineering, National Yang Ming Chiao Tung University, Taipei 112, Taiwan; yuhui8762@gmail.com

**Keywords:** distal radius fracture, dorsal plate, biomechanical test, four-point bending test

## Abstract

This study compares the absolute and relative stabilities of a novel hybrid dorsal double plating (HDDP) to the often-used dorsal double plating (DDP) under distal radius fracture. The “Y” shape profile with 1.6 mm HDDP thickness was obtained by combining weighted topology optimization and finite element (FE) analysis and fabricated using Ti6Al4V alloy to perform the experimental tests. Static and fatigue four-point bending testing for HDDP and straight L-plate DDP was carried out to obtain the corresponding proof load, strength, and stiffness and the endurance limit (passed at 1 × 10^6^ load cycles) based on the ASTM F382 testing protocol. Biomechanical fatigue tests were performed for HDDP and commercial DDP systems fixed on the composite Sawbone under physiological loads with axial loading, bending, and torsion to understand the relative stability in a standardized AO OTA 2R3A3.1 fracture model. The static four-point bending results showed that the corresponding average proof load values for HDDP and DDPs were 109.22 N and 47.36 N, that the bending strengths were 1911.29 N/mm and 1183.93 N/mm, and that the bending stiffnesses were 42.85 N/mm and 4.85 N/mm, respectively. The proof load, bending strength and bending stiffness of the HDDPs were all significantly higher than those of DDPs. The HDDP failure patterns were found around the fourth locking screw hole from the proximal site, while slight plate bending deformations without breaks were found for DDP. The endurance limit was 76.50 N (equal to torque 1338.75 N/mm) for HDDP and 37.89 N (equal to torque 947.20 N/mm) for DDP. The biomechanical fatigue test indicated that displacements under axial load, bending, and torsion showed no significant differences between the HDDP and DDP groups. This study concluded that the mechanical strength and endurance limit of the HDDP was superior to a commercial DDP straight plate in the four-point bending test. The stabilities on the artificial radius fractured system were equivalent for novel HDDP and commercial DDP under physiological loads in biomechanical fatigue tests.

## 1. Introduction

Distal radius fractures are the most common injuries encountered in orthopedics, which represent 17.5% of all fractures [1]. There are several options for the operative treatment of unstable fractures. Open reduction and plate fixation are the most common treatments for this type of injury. The surgical approach and plate selection should correlate with the fracture configuration. Although volar plating is more widely used, dorsal plating may be more reliably in preventing re-displacement in some instances of dorsally displaced metaphyseal fragments than volar plating.

Conventional dorsal plate application could result in wrist extensor tendon irritation and occasional extensor tendon rupture. The use of modern dorsal locking plates improved the clinical results and reduced the number of complications [2]. The three column concept introduced by Rikli and Regazzoni divides the distal radius and ulna into radial, intermediate, and ulnar columns [3]. Following the three column theory principles, by applying more than one low profile plate in the orthogonal plane with an angle of 50–70°, a multi-planar, load-sharing construct anatomically restores the articular surface while providing enough stability to allow immediate motion after surgery. AO/ASIF (Synthes, Paoli, PA, USA) 2.4-mm low-profile locking fragment-specific implants were then developed and are now widely used.

Although dorsal double-plating (DDP) has shown promising results, it still has some unsolved problems, including increased surgical time and cost. In addition, the fixation screws in the dorsal-volar direction of the T plate might interfere with the screw in the straight I plate radial–ulnar direction because of the limited distal fragment space [4]. Inadequate subchondral support due to fewer distal screw numbers is still a concern. An ideal fixation for the distal radius does not exist and optimal treatment remains a therapeutic challenge. To overcome these problems, a new dorsal locking plate, hybrid dorsal double plate (HDDP), has been designed based on weighted topology optimization and finite element analysis [5]. The HDDP is a “Y”-shaped plate with two ears on the top of the dorsal-radial and -ulnar sides to provide adequate support to the distal fragment. Multiple screws are inserted at the top dorsal-radial/-ulnar ears of the HDDP to enhance the stability. Early numerical biomechanical evaluation reported promising results. From the FE analysis [5], the stress concertation was noted in the DDP group. The DDP intermediate and styloid plate stress concentrations were found at the middle of the plates (around the fracture sites). This might increase the risk for implant failure. On the other hand, stress concentration was not significant in the HDDP group. By changing the shape of the plate, the stress on the plate can be redistributed, so the risk for stress concertation could be diminished. However, clinical data are still awaited.

This study aimed to compare the absolute and relative stability of the newly designed HDDP with the often-used DDP under in vitro fatigue four-point bending and biomechanical tests in an anatomically fashioned synthetic bone model. The hypothesis of this article is that the novel plate shows equivalent or superior mechanical properties in load-bearing capacity and endurance.

## 2. Materials and Methods

### 2.1. HDDP Design and Manufacture

**Figure 1:**
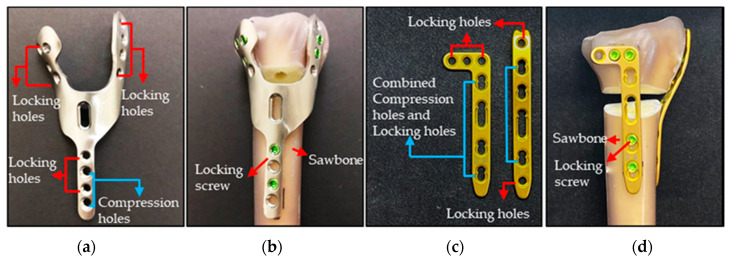
(**a**) Profile of the novel HDDP presented a “Y” shape with 1.6 mm thickness, fabricated using Ti6Al4V alloy. (**b**) The HDDP was fixed onto one of the radius Sawbones using self-tapping 2.4 mm locking screws bicortically in the shaft and unicortically in the distal end. (**c**) The dorsal double-plating (DDP) consists of one “L” shape plate and one straight plate with 1.6 mm thickness, fabricated using Ti6CP alloy. (**d**) The DDP was fixed onto one of the radius Sawbones using self-tapping 2.4 mm locking screws bicortically in the shaft and unicortically in the distal end around the fixation screws.

The novel HDDP profile presents a “Y” shape with 1.6 mm thickness, obtained by combining weighted topology optimization and finite element (FE) analysis under six fracture models with 50%, 30%, and 20% weights of the joint subjected to axial, bending, and torsion moments, respectively, as presented in our previous study [5] (Figure 1a). The novel HDDP has been approved with the best optimal structural strength when compared with the DDP approach and the novel HDDP achieves the objective of being light weight with a single-wound surgical approach. The two ears’ geometric features on the distal top of the dorsal-radial and -ulnar sides with eight locking screws and no screw interferences were designed to provide adequate support to distal articular comminuted fractures. The HDDP can be placed on the dorsal site of the distal radius through the standard dorsal approach. The numerical biomechanical simulation results indicated that the novel HDDP demonstrated better resistances to functional loads and provided sufficient screw fixation at the articular surface than those with DDP (Figure 1c), regardless of the bone fracture type.

The physical model of novel HDDP is composed of Ti6Al4V alloy (Figure 1a) and fabricated using an ISO13485 quality management systems company (Microware Precision Technology Co., Ltd., Taichung City, Taiwan) for the following fatigue four-point bending and biomechanical tests.

### 2.2. Four-Point Bending Mechanical Testing

To provide a comprehensive mechanical strength reference for bone plates used in the surgical internal fixation of the skeletal system, four-point bending tests were carried out for novel HDDP and commercial bone plate according to the American Society for Testing and Materials (ASTM) protocols (ASTM F382-17), using the Instron E10000 testing machine (INSTRON, Canton, MA, USA). The commercial straight L-plate titanium locking compression double plate fragment-specific system (LCP L/I 2.4, Deupy Synthes, Synthes GmbH, Eimattstrasse, Switzerland) was set as the comparison group with HDDP in the static and fatigue tests.

**Figure 2:**
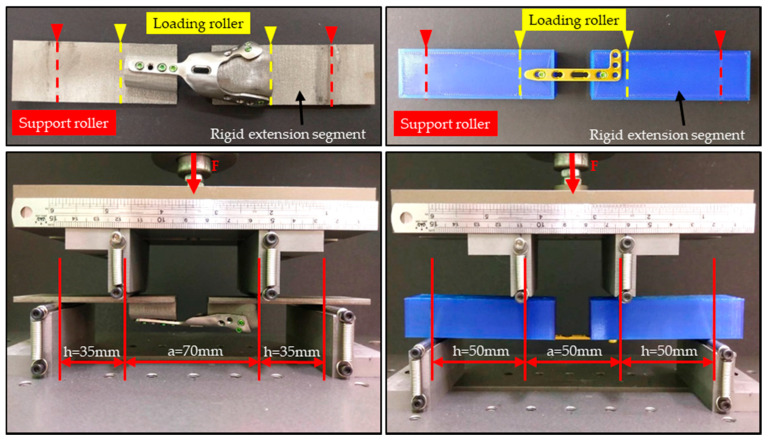
The HDDP (left part) and L-plate (right part) were fixed onto the corresponding rigid extension segments; the loading rollers made contact with the rigid extension segments of the test setup during the test.

Static testing was performed first to obtain the proof load, strength, and stiffness of the samples. The rigid extension segments used to effectively lengthen the bone plate due to the HDDP do not have a symmetrical section, and the L-plate does not have a sufficiently long symmetrical section. The geometry profile at the proximal and distal sides of the rigid extension segments for the HDDP were designed according to their corresponding geometric features of the contact bone plate and fabricated using a metal 3D printer (AM 400, Renishaw, Gloucestershire, UK). The HDDP and L-plate were fixed onto the corresponding rigid extension segments and the loading rollers contact the rigid extension segments of the test setup during the test (Figure 2).

Center span (a) is the distance between the loading rollers and 70 mm for the HDDP and 50 mm for the L-plate test samples. Additionally, the loading span distance (h) is the distance between the loading roller and nearest support roller. The corresponding distances for the HDDP and L-plate samples were 35 mm and 50 mm, respectively (Figure 2). Each group had three samples and were placed on the four-point bending test clamp to load at a cross-head rate of 0.05 mm/s until failure occurred. The proof load (P), plate deflection, and force-displacement diagram were collected from each test, and the corresponding bending strength, bending stiffness (K), and bending structural stiffness were calculated. The failure pattern of each sample was visually examined to assess the failure mechanism.

According to the ASTM F382 standard test method, bending stiffness (K) was determined by measuring the linear portion slope of the load–displacement curve. The bending structural stiffness (EI) was derived from the four-point bending load apparatus as follows:

Bending Structural Stiffness = EI = [(2h + 3a) kh^2/12],(1)

Bending strength was calculated using the following formula:

Bending Strength = Ph/2,(2)

where P is the proof load at the intersection line of a 0.2% offset from the linear portion of the load–displacement curve in the load versus load–displacement curve.

The fatigue test was performed according to the result of the proof load obtained from the static four-point bending test. The fatigue tests were run using a sinusoidal cyclic load waveform at a constant frequency of 5 Hz in the four-point bending apparatus. Fatigue testing was considered complete when either the limit of one million cycles was reached or when failure occurred, either through cracking or plastic deformation, resulting in a displacement greater than twice the initial displacement. There were 90%, 80%, and 70% of the proof load values tested in the HDDP group and 95%, 90% and 80% of the proof load values tested in the L-plate group. Three samples were investigated in each load condition.

### 2.3. Biomechanical Fatigue Test

In order to more closely simulate the bone plate load under physiological conditions, biomechanical fatigue tests were performed to compare the mechanical responses between the newly developed HDDP and commercial DDP systems using a composite Sawbone (Sawbones; Pacific Research Laboratories Inc., Vashon Island, WA, USA). Sawbone has been previously proven to have similar mechanical properties to cadaver bone [6]. As the synthetic bone is produced industrially, its availability, comparability, and reproducibility are superior to natural bone. 

Eighteen Sawbone radii were prepared and cut at the proximal third of the bone, giving a length of 230 mm to allow for standardized embedding in an epoxy resin block. A standardized AO OTA 2R3A3.1 fracture model was created with a 10 mm dorsal defect, 20 mm distal to the articular surface to simulate the extra-articular fracture. Our novel HDDP and commercial DDP were fixed, respectively, onto nine of the Sawbones radii using self-tapping 2.4 mm locking screws bicortically in the shaft and unicortically in the distal end (Figure 1b,d).

**Figure 3:**
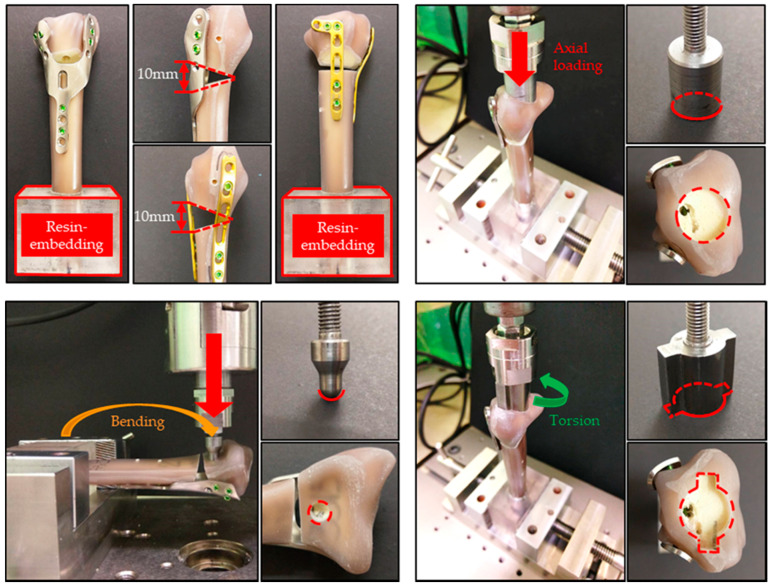
The HDDP and DDP were fixed onto the fractured Sawbone radius (upper-left part). The fractured Sawbone radius was embedded with resin for stable fixation on the vise. Three kinds of loads (axial load, bending, and torsion at the upper-right, lower-left, and lower-right parts, respectively) were applied to the distal radial surface through the corresponding specific loading devices.

The HDDP and DDP groups were divided into three subgroups, including axial load, bending, and torsion. All of the biomechanical fatigue tests were performed using the Instron 10,000 testing machine with 20,000 load cycles to represent the upper end of the estimated physiological loads seen over the usual 6-month healing time for this injury [7,8,9,10,11]. The specimens were subjected to all loads oscillated between 15 N and 150 N at 5 Hz for axial load, 5.5 N to a maximum of 55 N at 5 Hz for bending, and −1 N-m to 1 N-m at 5 Hz for torsion. The maximum applied load represents the upper end of the estimated physiological forces with wrist motion [7,10,12,13]. Three kinds of loads were respectively applied to the distal radial surface through the corresponding specific loading devices (Figure 3). One-way analyses for axial displacement under axial load, lateral displacement under bending, and rotational degree under torsion between HDDP and DDP fixations were performed after the fatigue tests to understand the statistical significance.

## 3. Results

**Figure 4:**
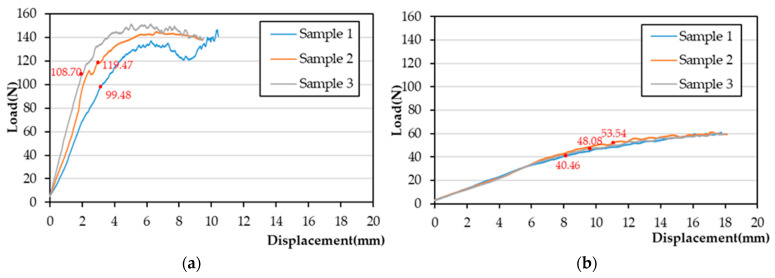
Static four-point bending test load displacement diagrams for HDDP and DDP. The red dots indicate the proof load (**a**) for HDDP and (**b**) for DDP.

In the static four-point bending test, the corresponding values for HDDP and DDPs of the average proof load were 109.22 ± 8.17 N and 47.36 ± 5.36 N (Figure 4), the bending strengths were, 1911.29 ± 142.93 N/mm and 1183.93 ± 134.09 N/mm, and the bending stiffnesses were, 42.85 ± 9.21 N/mm and 4.85 ± 0.20 N/mm, respectively. The proof load, bending strength, and bending stiffness of the HDDPs were all significantly higher than those for DDPs.

**Figure 5:**
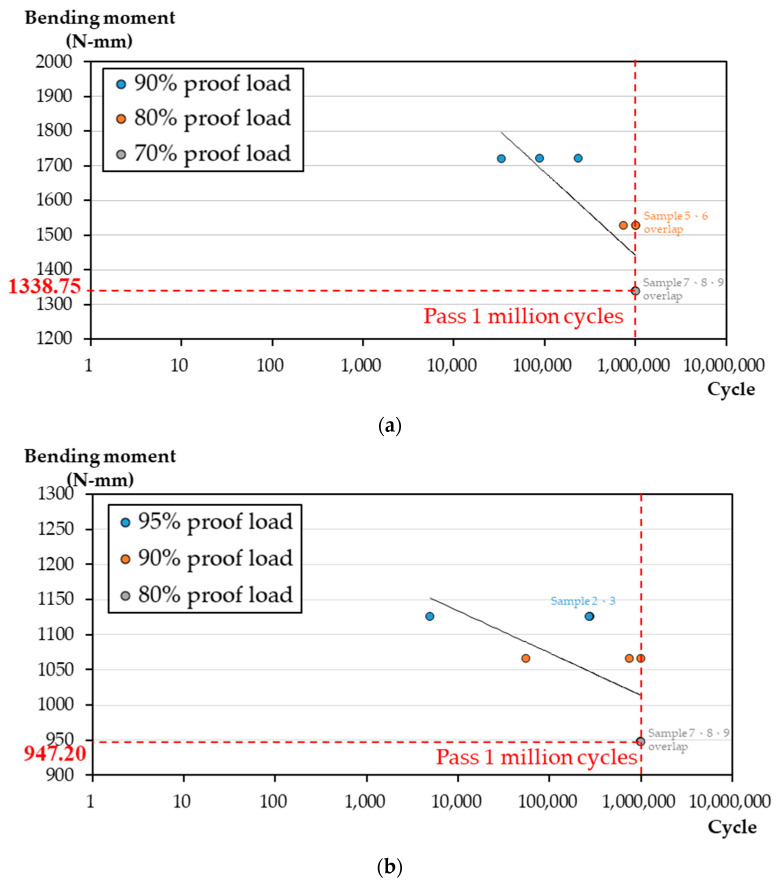
The four-point bending fatigue test M-N diagrams for HDDP and DDP and their corresponding endurance limit (**a**) for HDDP and (**b**) for DDP, i.e., past one-million cycle load.

In the fatigue four-point bending test, fatigue M-N diagrams of the HDDP and DDP groups are plotted in Figure 5. The HDDP past one million cycle load fatigue limit was 76.50 N (equal to torque 1338.75 N/mm, 70% of the proof load) (Figure 5a), and the corresponding DDP group value was 37.89 N (equal to torque 947.20 N/mm, 80% proof load) (Figure 5b). All three samples failed in 90% of the proof load tests (98.3 N, equal to 1720.25 N/mm), two samples withstood one million cycles, and one sample failed in 80% of the proof load tests (87.4 N, equal to 1529.5 N/mm) for the HDDP group. In contrast with DDP, all three samples failed in 95% of the proof load tests (45.00 N, equal to 1125.00 N/mm), one sample withstood one million cycles and two samples failed in 90% of the proof load tests (42.62 N, equal to 1065.0 N/mm) of the DDP group (Table 1).

**Figure 6:**
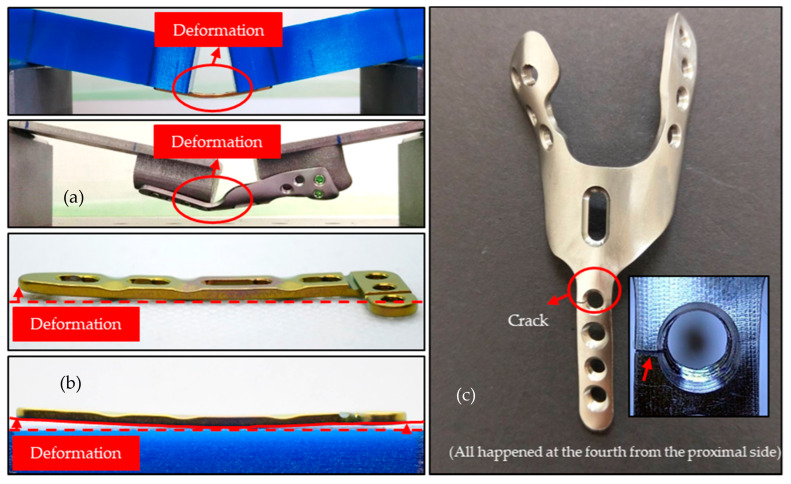
HDDP and DDP group case failure patterns. Deformations of (**a**) HDDP; (**b**) DDP and (**c**) cracks of HDDP.

The failure patterns in the HDDP and DDP groups were different. Plate cracks were found around the fourth locking screw hole from the proximal site, which is also the most distally locking screw hole for radial shaft fixation for the HDDP group. However, slight plate bending deformations without breaks were found in central areas of the plate for the DDP group (Figure 6).

**Figure 7:**
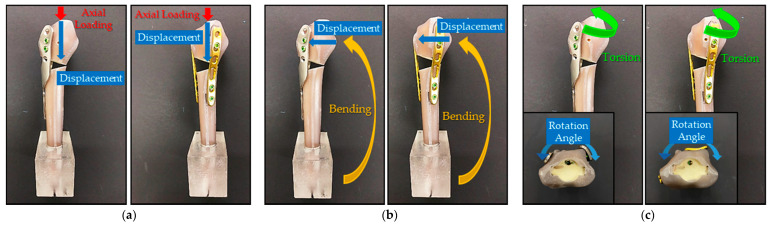
Illustration of the biomechanical fatigue test displacements under (**a**) axial load, (**b**) bending, and (**c**) torsion.

The biomechanical fatigue test result showed that average axial displacement values under axial load for the HDDP and DDP groups were 0.4218 mm and 0.5162 mm, respectively. Corresponding HDDP and DDP group values for lateral displacement under bending were 1.7623 mm and 2.2045 mm; rotational degrees under torsion were 1.9625° and 1.9736°, respectively (Figure 7). All variations of obtained values under axial load, bending, and torsion showed no significant differences between the HDDP and DDP groups (Table 2).

## 4. Discussion

A distal radius fracture is a common fracture. The treatment goals are anatomical reduction, stable fixation, and early mobilization. Obtaining and maintaining an anatomical reduction would lead to a good clinical outcome. For this reason, open reduction and internal fixation have become the most common treatments for these injuries. Good outcomes have been reported with dorsal plates that can buttress the dorsal cortex comminution and maintain distal fragment dorsal displacement reduction [7,9,14]. It can be technically demanding to place certain plates on the dorsal surface of the distal radius because of the irregularity. In addition, there is limited soft tissue between the skin and bone surface, which may result in symptomatic fixation plate prominence. Sometimes, extensor tendon irritation or rupture occur due to the tendon making direct contact with a prominent dorsal plate or screws [15,16,17]. In order to avoid these complications, it is suggested that dorsal plates be low profile. As the indications for operative treatment of displaced distal radius fractures increase, a solid and reliable implant is indispensable. Repetitive axial, bending, and torsion forces accumulated within a distal radius plate over time may lead to plate failure if bone healing is delayed [18]. For this reason, it is important to know the biomechanical characteristics of the available plates.

A novel HDDP is therefore proposed with a “Y”-shaped plate and two ears on the top of the dorsal-radial and -ulnar sides to provide adequate support to the distal fragment. Multiple screws are inserted at the top dorsal-radial/-ulnar ears of the HDDP to enhance stability for treating comminuted or osteoporotic fractures. Additionally, minimally invasive techniques for plate osteosynthesis can be applied with the HDDP due to its advantages in minimizing soft-tissue damage and in preserving the blood supply to the fracture site. The pre-shaped anatomical design of the plate does not require implant adaptation to bone geometry. Due to the low-profile plate design, it is possible to insert it through a small incision. However, in vitro biomechanical experiments need to be performed to verify the mechanical feasibility of the HDDP to supplement the previous results from FE analysis. In order to meet the FDA’s requirements for bone plate functional testing and the feasibility of future clinical use, four-point bending mechanical testing and fatigue biomechanical testing were planned under physiological load conditions.

Four-point bending testing is a specification and test method that provides a comprehensive reference for bone plates used in skeletal system surgical internal fixation. The standard establishes consistent methods to classify and define the geometric characteristics and performance characteristics of bone plates. It is not the intention of the standard to define levels of performance or case-specific clinical performance for bone plates, as insufficient knowledge is available to predict the consequences from their use in individual patients for specific activities of daily living [19]. The static test result showed that the proof load, bending strength, and bending stiffness of the HDDPs were all significantly higher than those of DDPs. This might be due to the fact that the material yield strength of HDDP (titanium alloy) and the second moment of inertia of the relative plate cross-sectional area were higher than that of DDP (pure titanium). In the fatigue endurance limit comparison, the bending strength values were compared between HDDP and DDPs, instead of proof load, because of the HDDP complex geometry rather than the DPP flat plate. Obviously, the HDDP endurance limit (1338.75 N/mm) was higher than that of DDP (947.20 N/mm). This result shows that substantial functional test equivalence between HDDP and DDP under the same clinical indication can meet FDA requirements for a safe and effective legally marketed device. Moreover, the majority of the failures in HDDP were plate-screw interface failure, whereas DDP displayed more plate deformation. The failure mechanisms determined here at least partially explain the clinically observed plate failure problem. The Synthes dorsal plates showed signs of plastic deformation already at loads below 50 N in the single static test. It is conceivable that everyday situations might lead to plate dorsal and volar bending, which might lead to plate failure in the long run. A study pointed out that loads transmitted to the distal radius are about 50 N for each 10 N of grip forces [12]. However, the HDDP was relatively safe because its proof load was nearly twice that of Synthes dorsal plates. 

Fatigue biomechanical testing using artificial Sawbone under physiological loads was performed to understand the mechanical behaviors for HDDP and commercial plates. This is because the loads close to the fracture area are always changing and repeating when active finger mobilizations are performed during the acute healing period. Cyclic loading rather than single loading was included in these experiments. Artificial Sawbone was used because the size and shape are easier to standardize, making the mechanical evaluation and comparison more reliable. As suggested in a previous publication, we tested for torsional and axial compression as well as bending [13]. The loads used in this study were designed to be comparable with the loads applied to the distal radius during early postoperative hand and wrist mobilization. Loads transmitted to the distal radius were about 50 N for each 10 N of grip forces with various hand positions and radius lengths [12]. Although the compression forces on the distal radius in vivo have not yet been clearly defined, several studies suggested that compressive forces created by light, active wrist motion do not exceed 100 N. Combined wrist and digit forces of motion do not exceed more than 250 N [10,20,21,22]. Therefore, in our study, the specimens were performed under load control to a force of 150 N for axial compression, 55 N for bending (about 11,000 N/mm bending moment), and 10,000 N/mm for torsion.

However, our results showed that there were no significant differences for maximum axial, bending, and torsional displacements between the HDDP and commercial DDP constructs. This result indicates that the HDDP dorsal plate biomechanical performance was as safe as the commercial DDP product. However, further observation showed that the average HDDP displacements in axial and bending were smaller than those for the commercial DDP, while the relative torsional value was higher. This result implied that HDDP use should try to avoid forearm pronation and supination after surgery. In general, the novel HDDP should be able to further undergo clinical trials based on biomechanical testing results, less surgical time, and smaller surgical wound. 

There were limitations in this study, including one simple fracture (2R3A3.1) model and independent physical loads that do not interfere with each other being only considered in our biomechanical fatigue testing. More complex fractures and cadaver samples may be taken into account in further in vitro testing to assess the safety and effectiveness of the HDDP in treating selected distal radius fractures. Furthermore, this study focused on the macroscopic stability comparison of fixations but lacked the microcosmic strain analysis of fragment interfaces. In considering healing objectives, a continuous principal strain analysis comparison study can be executed in the future.

## 5. Conclusions

This study showed the mechanical superiority of the HDDP compared with a regular straight dorsal plate in a four-point bending test. The fatigue biomechanical testing result under axial load, bending, and torsion displayed no significant difference for corresponding displacements between the HDDP and commercial DDP constructs in extra-articular distal radius fractures. However, everyday movements are complex and bone-plate construct stability in multiple directions can contribute to fixation failure. Thus, further clinical studies are required to investigate the implications of HDDP. The current investigation was the first to biomechanically compare HDDP fixation with dorsal double plating of distal radius fractures, providing a quantitative assessment of an alternative fixation strategy. We recommend the HDDP for clinical use, especially when treating comminuted or osteoporotic fractures based on our biomechanical results and in agreement with our clinical experience.

## Figures and Tables

**Table 1:** Fatigue four-point bending test results for HDDP and DDP.

	% of Proof Load	Load (N) (Equal to Torque (N/mm))	Sample Number	Cycle	Result	Temp/Humidity (°C/%)
HDDP	90%	9.83~98.30 (172.03~1720.25)	1	33,000	Failure	22 °C/70%
			2	89,000	Failure	22 °C/70%
			3	237,000	Failure	22 °C/70%
	80%	8.74~87.40 (152.95~1529.5)	4	729,000	Failure	22 °C/70%
			5	1,000,000	Pass	22 °C/70%
			6	1,000,000	Pass	22 °C/70%
	70%	7.65~76.50 (133.88~1338.75)	7	1,000,000	Pass	22 °C/70%
			8	1,000,000	Pass	22 °C/70%
			9	1,000,000	Pass	22 °C/70%
Synthes Dorsal Plate	95%	4.50~45.00 (112.50~1125.00)	1	5000	Failure	22 °C/70%
			2	281,921	Failure	22 °C/70%
			3	272,272	Failure	22 °C/70%
	90%	2.26~42.62 (106.56~1065.60)	4	54,998	Failure	22 °C/70%
			5	1,000,000	Pass	22 °C/70%
			6	739,758	Failure	22 °C/70%
	80%	2.49~37.89 (94.72~947.20)	7	1,000,000	Pass	22 °C/70%
			8	1,000,000	Pass	22 °C/70%
			9	1,000,000	Pass	22 °C/70%

**Table 2:** Biomechanical fatigue test displacement results under axial load, bending, and torsion.

	Axial Loading Maximum Axial Displacement (mm)		Bending Maximum Bending Displacement (mm)		Torsion Maximum Angular Displacement (Degree)	
	HDDP	DDP	HDDP	DDP	HDDP	DDP
Sample 1	0.3797	0.8941	1.8203	1.4245	2.3499	2.7659
Sample 2	0.3818	0.3076	1.8911	3.2378	1.3718	1.5019
Sample 3	0.5039	0.3471	1.5754	1.4113	2.1659	1.2032
Average Value (Standard deviation)	0.4218 (0.0581)	0.5162 (0.2676)	1.7623 (0.1353)	2.0245 (0.8579)	1.9625 (0.4244)	1.8237 (0.6774)
*t*-test (α = 0.05)	*p*-value = 0.3370 > 0.05 no significant difference		*p*-value = 0.3555 > 0.05 no significant difference		*p*-value = 0.4109 > 0.05 no significant difference	

## Data Availability

Not applicable.
